# Perioperative ABO Blood Group Isoagglutinin Titer and the Risk of Acute Kidney Injury after ABO-Incompatible Living Donor Liver Transplantation

**DOI:** 10.3390/jcm10081679

**Published:** 2021-04-14

**Authors:** Hyeyeon Cho, Jinyoung Bae, Hyun-Kyu Yoon, Ho-Jin Lee, Seong-Mi Yang, Suk Hyung Choe, Chul-Woo Jung, Kyung-Suk Suh, Won Ho Kim

**Affiliations:** 1 Department of Anesthesiology and Pain medicine, Seoul National University Hospital, Seoul National University College of Medicine, #101 Daehak-ro, Jongno-GU, Seoul 03080, Korea; bdbd7799@gmail.com (H.C.); baejy88@gmail.com (J.B.); hyunkyu18@gmail.com (H.-K.Y.); zenerdiode03@gmail.com (H.-J.L.); seongmi.yang@gmail.com (S.-M.Y.); paulchoe17@snu.ac.kr (S.H.C.); jungcwoo@gmail.com (C.-W.J.); 2 Department of Surgery, Seoul National University Hospital, Seoul National University College of Medicine, #101 Daehak-ro, Jongno-GU, Seoul 03080, Korea; kssuh@snu.ac.kr

**Keywords:** liver transplantation, living donor, ABO blood-group system, isoagglutinin, anti-ABO blood type antibody, acute kidney injury, therapeutic plasma exchange

## Abstract

For ABO-incompatible liver transplantation (ABO-i LT), therapeutic plasma exchange (TPE) is performed preoperatively to reduce the isoagglutinin titer of anti-ABO blood type antibodies. We evaluated whether perioperative high isoagglutinin titer is associated with postoperative risk of acute kidney injury (AKI). In 130 cases of ABO-i LT, we collected immunoglobulin (Ig) G and Ig M isoagglutinin titers of baseline, pre-LT, and postoperative peak values. These values were compared between the patients with and without postoperative AKI. Multivariable logistic regression analysis was used to evaluate the association between perioperative isoagglutinin titers and postoperative AKI. Clinical and graft-related outcomes were compared between high and low baseline and postoperative peak isoagglutinin groups. The incidence of AKI was 42.3%. Preoperative baseline and postoperative peak isoagglutinin titers of both Ig M and Ig G were significantly higher in the patients with AKI than those without AKI. Multivariable logistic regression analysis showed that preoperative baseline and postoperative peak Ig M isoagglutinin titers were significantly associated with the risk of AKI (baseline: odds ratio 1.06, 95% confidence interval 1.02 to 1.09; postoperative peak: odds ratio 1.08, 95% confidence interval 1.04 to 1.13). Cubic spline function curves show a positive relationship between the baseline and postoperative peak isoagglutinin titers and the risk of AKI. Clinical outcomes other than AKI were not significantly different according to the baseline and postoperative peak isoagglutinin titers. Preoperative high initial and postoperative peak Ig M isoagglutinin titers were significantly associated with the development of AKI. As the causal relationship between high isoagglutinin titers and risk of AKI is unclear, the high baseline and postoperative isoagglutinin titers could be used simply as a warning sign for the risk of AKI after liver transplantation.

## 1. Introduction

The incidence of acute kidney injury (AKI) after liver transplantation has been reported to be up to 68% [1,2,3,4,5,6,7,8,9,10,11]. AKI is regarded to be clinically important because it is associated with poor graft survival, the development of chronic kidney disease [12,13], and increased mortality [3,4,7,14,15].

In this era of donor organ shortage for end-stage liver disease, ABO-incompatible liver transplantation (ABO-i LT) is a good therapeutic choice [16]. Graft outcomes after ABO-i LT significantly improved after adopting desensitized preoperative protocols using rituximab and therapeutic plasma exchange (TPE). The graft survival after ABO-i LT is currently comparable to ABO-compatible liver transplantation [16,17,18,19,20]. However, the incidence of AKI after ABO-i LT has still been reported to be higher than ABO-compatible LT [9]. Therefore, it would be important to investigate the potentially modifiable risk factors of AKI in ABO-i LT to reduce the high incidence of postoperative AKI. 

In ABO-i LT, TPE is repeated until the isoagglutinin titer of anti-ABO blood type antibody is reduced to a target level of 1:16 or less. The isoagglutinin titer can be associated with the risk of AKI because the high baseline isoagglutinin titer may require multiple TPEs accompanied by large amounts of fresh frozen plasma transfusion. Repeated large amounts of transfusion can lead to systemic inflammatory response, which can result in AKI. Also, a high baseline or postoperative isoagglutinin titer can lead to antibody-mediated graft rejection, and the resulting early allograft dysfunction can be associated with major organ dysfunction, including of the kidneys.

Initial range of preoperative immunoglobulin (Ig) M and Ig G isoagglutinin titer varies among patients. There is no significant difference between the initial high and low Ig M and Ig G isoagglutinin groups regarding transplantation outcomes, including laboratory findings and incidence of surgical complications [17], when adequate TPE protocols and immunosuppressive agents are used and target levels of isoagglutinin are achieved. However, intrahepatic biliary complications and graft necrosis are closely associated with peak titer of pre- and post-LT Ig G or Ig M isoagglutinin [21]. Thus, most of the current interventions to improve the outcomes of ABO-i LT have are used toward the reduction of anti-ABO blood type antibodies to a safe low level before transplantation. However, it is still unknown whether the isoagglutinin titer could affect the postoperative incidence of AKI. The relationship between initial baseline isoagglutinin titers and the risk of post-transplant AKI has not been reported. It is unclear whether baseline Ig G or Ig M antibodies are more important for that association in ABO-i LT. In addition, the association between the postoperative elevation of isoagglutinin titer and AKI has not been reported.

To evaluate these associations, in this retrospective observational study we investigated whether the risk of AKI is associated with perioperative Ig M and Ig G isoagglutinin titer. We hypothesized that the incidence of AKI is higher in patients with high initial baseline or postoperative peak Ig G and Ig M isoagglutinin titer and that there is a dose-response association between the perioperative isoagglutinin titer and the risk of AKI.

## 2. Materials and Methods

### 2.1. Study Design

The institutional review board (IRB) of Seoul National University Hospital approved our retrospective observational study (2101-031-1185). We reviewed the electronic medical records of 134 consecutive cases of patients who underwent elective ABO-i living donor LT at our institution between January 2008 and December 2020. The requirement for written informed consent was waived by the IRB, considering our study’s retrospective design. We excluded the patients with baseline chronic kidney disease stage 3a (<60 mL/min/1.73 m^2^) or higher (*n* = 2) and with preoperative renal dysfunction, including hepatorenal syndrome type 1 (*n* = 2). We analyzed the data of the remaining 130 cases.

### 2.2. Surgical Technique, Anesthesia, and Desensitization Protocols

Anesthesia for liver transplantation surgery was maintained with propofol with remifentanil. Volume-controlled ventilation was maintained with a tidal volume of 6–8 mL/kg. Arterial-line catheters were inserted into the radial and femoral arteries. Ephedrine, phenylephrine and continuous infusion of norepinephrine and/or epinephrine were used to treat hypotension, guided by the monitored cardiac output, systemic vascular resistance and mixed venous oxygen saturation. Packed red blood cells (pRBCs) of the same blood type of recipient were transfused during surgery when hematocrit fell below 20%. Fresh frozen plasma (FFP) and platelet concentrate of the blood type AB were transfused. A histidine–tryptophan–ketoglutarate solution was used for donor graft. The piggyback technique was used to anastomose donor vessels and the graft. The position of venous clamp was adjusted to maintain adequate cardiac preload by surgeons. Venovenous bypass was not used. After the hepatic vein and portal vein anastomosis were completed, the liver graft was reperfused via the consecutive release of the clamping over hepatic and portal veins. The hepatic artery and bile duct were anastomosed in an end-to-end fashion.

All patients were administered an intravenous dose of rituximab (300–375 mg/m^2^ body surface area) at 2–3 weeks before surgery. All ABO-i LT recipients’ anti-ABO isoagglutinin titers were assessed at admission, at each round of TPE, on the day before the surgery, and until the second post-transplant week. The timing and frequency of assessment were readjusted depending on the isoagglutinin level. For plasma exchange, blood type AB fresh frozen plasma was used. TPE was repetitively performed to achieve an isoagglutinin titer of Ig M anti-ABO blood type antibody of 1:16 or less before the surgery. If the isoagglutinin titer just before LT was higher than 1:16, postoperative high dose intravenous immunoglobulin (IVIG) at 0.8 g/kg/day was administered during the five postoperative days and simultaneous splenectomy was performed during transplantation surgery. TPE was repeated postoperatively if isoagglutinin titer rebound (≥1:32) occurred or there were increasing slopes of isoagglutinin titers or serum creatinine or clinical sign of acute antibody-mediated rejection.

During the anhepatic period, intravenous 10 mg/kg methylprednisolone was administered just before graft reperfusion. This was switched to oral 0.5 mg/kg/day methylprednisolone, tapering over 3 months after ABO-i LT [18]. Immunosuppression after ABO-i LT comprised triple therapy of corticosteroid, tacrolimus and mycophenolate mofetil (0.5–1.5 g/day) [18,22].

### 2.3. Data Collection

Patient demographics or baseline characteristics associated with postoperative renal dysfunction were collected [2,3,6,7,23,24,25,26]. Preoperative Child-Turcotte-Pugh classification and the Model for End-stage Liver Disease (MELD) score were determined for all patients [27]. History of diabetes mellitus, hypertension, warm and cold ischemic time, pre-, intra- and postoperative transfusion amount, postreperfusion syndrome, and the amount of crystalloid and colloid administration were collected. Transfusion amount was collected from the initiation of TPE until the patient discharge.

Postreperfusion syndrome is defined as a 30% or greater decrease in mean blood pressure at least for 1 min within 5 min of portal vein reperfusion compared with the baseline pressure observed immediately before reperfusion [2,8]. Isoagglutinin titers of Ig G and Ig M anti-blood-type antibodies were collected during pre- and postoperative periods. Daily titers were collected while TPE was performed, and postoperative titers were collected during the first two weeks post operation. For blood type O recipients whose donor was blood type AB, isoagglutinin titers of both anti-A and anti-B were collected, and we used the higher value for our data analysis. Titer reduction rate (TRR), which represents the mean titer reduction per one session of TPE, was calculated as follows: TRR = (baseline Isoagglutinin titer step before initiation of TPE—last isoagglutinin titer before transplantation/number of pre-liver transplantation plasma exchange [28]. Postoperatively, the daily tacrolimus trough level was collected during the first postoperative week.

Our primary outcome was postoperative AKI defined by Kidney Disease Improving Global Outcomes (KDIGO) criteria [29], which was validated in liver transplantation [1,13]. AKI was diagnosed based on the post-transplant increase in serum creatinine (stage 1: ≥0.3 mg/dL increase within 48 h or 1.5–1.9; stage 2: 2–2.9; stage 3: more than 3-fold increase of baseline, respectively within the first seven days after transplantation). The baseline serum creatinine was determined as the most recent value measured before surgery. Secondary outcomes included postoperative laboratory findings of total bilirubin, aspartate aminotransferase, alanine aminotransferase, prothrombin time: international normalized ratio (PT:INR), serum creatinine, and estimated glomerular filtration rate as well as the incidence of antibody-mediated rejection, acute cellular rejection, surgical site infection and biliary and vascular complication. We also collected the incidence of early allograft dysfunction, defined by the presence of one or more of the following: alanine or aspartate aminotransferase >2000 IU/L or PT:INR >1.6 or total bilirubin >10 mg/dL within the first 7 days after transplantation. Other postoperative clinical outcome variables included the incidence of post-transplant hemodialysis, length of hospital stay and intensive care unit stay.

### 2.4. Statistical Analysis

We used STATA/MP version 15.1 (StataCorp, College Station, TX, USA) and SPSS software version 25.0 (IBM Corp., Armonk, NY, USA) to analyze the data. Medcalc Statistical Software version 18.6 (MedCalc Software bvba, Ostend, Belgium) was used to draw the distribution plot; *p* < 0.05 was considered statistically significant for all analyses. Bonferroni correction was used to adjust the comparisons for multiple time points. The Kolmogorov-Smirnov test was used to determine the normal distribution of our data. We used Fisher’s exact test or chi-square test to compare the categorized variables. Continuous variables were compared using the Mann-Whitney U test. We found missing data in <5% of records. We replaced missing values of continuous variables by the age- and sex-specific median values and assigned the most frequent age- and sex-specific values for missing categorized data.

To evaluate the multivariable association between the perioperative parameters, including isoagglutinin titers and tacrolimus trough level, and the risk of AKI, we performed multivariable logistic regression analysis. Potential predictors that were reported to be associated with post-transplant AKI were considered in the logistic regression model [2,3,6,7,23,24,25]. We used a backward Wald variable selection process with a significance criterion of 0.20. The following variables were used as covariates: age, body-mass index, sex, history of diabetes mellitus, hypertension, MELD score, causes of liver cirrhosis including hepatitis B virus, hepatitis C virus, hepatocellular carcinoma, alcoholism, preoperative hemoglobin concentration, preoperative serum creatinine, operation time, graft ischemic time and occurrence of postreperfusion syndrome. We evaluated the performance of our multivariable logistic regression model by the area under the receiver-operating characteristic curve and Negelkerke’s R^2^. We evaluated the calibration of our model by Hosmer-Lemeshow goodness-of-fit test.

As a sensitivity analysis to evaluate the time-dependent association between the isoagglutinin titers at three time points, tacrolimus levels and postoperative risk of AKI, the multivariable generalized estimating equation model was used to account for multiple measurements per patient and to assess the effects of factors predictive of AKI.

For descriptive statistics, the distribution of the initial baseline isoagglutinin titers of the Ig G and Ig M anti-ABO blood type antibodies were depicted. According to this distribution, patients were divided into two groups according to their initial baseline Ig M titers (high: Ig M ≥ 1:128; low: Ig M ≤ 1:64). Patients were also divided into two groups according to their postoperative peak Ig M titer (high: Ig M ≥ 1:32; low Ig M ≤ 1:16). Then, we compared our primary and secondary outcomes between high and low initial baseline and postoperative peak isoagglutinin Ig M titer to evaluate whether there was a significant difference in clinical outcomes between these high and low groups. Ig M titer rather than Ig G titer was chosen because our multivariable analysis of AKI showed that the effect size of Ig M titer was greater than Ig G titer. Postoperative daily tacrolimus blood trough levels were compared between the patients with and without AKI to evaluate whether there was a significant difference in the level between the two groups.

Cubic spline function curves were drawn to evaluate the multivariable-adjusted relationship between the isoagglutinin titer and the risk of AKI. Ig G and Ig M isoagglutinin titers of preoperative baseline and pre-LT values were analyzed. Postoperative daily laboratory values of total bilirubin, PT:INR, aspartate aminotransferase (AST), alanine aminotransferase (ALT), serum creatinine, and estimated glomerular filtration rate values were compared between high and low initial postoperative peak Ig M isoagglutinin titers to evaluate the association between the postoperative elevation of isoagglutinin titer and graft and renal function. Estimated glomerular filtration rate was calculated based on the Modification of Diet in Renal Disease (MDRD) formula [30].

Kaplan-Meier survival curve analysis was performed to evaluate the association between the isoagglutinin titer and long-term survival. Graft survival or all-cause mortality was compared between high and low baseline and postoperative peak isoagglutinin titer groups. The log-rank test was used to compare survival between the isoagglutinin groups.

## 3. Results

During the first seven postoperative days, AKI, determined by KDIGO criteria, occurred in 55 (42.3%) of the patients undergoing ABO-i LT (stage 1, *n* = 34, 26.1%; stage 2 or 3, *n* = 21, 16.2%). Patient demographics and characteristics were compared between patients with high and low initial baseline Ig M isoagglutinin titers (Table 1). Supplemental Table S1 shows the comparison of patient demographics and characteristics between patients with and without AKI.

**Figure 1:**
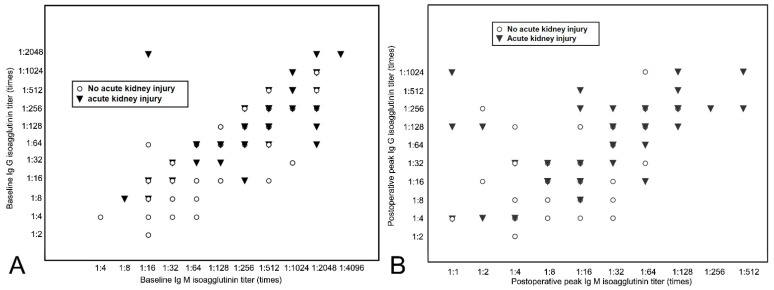
Distribution of the initial baseline (**A**) and postoperative peak (**B**) Ig G and Ig M isoagglutinin titers of the patients (*n* = 130). Ig = immunoglobulin. Data symbol is depicted where there is a case with each specific Ig G and Ig M titer.

Figure 1 shows the distribution of the initial baseline and postoperative peak Ig G and Ig M isoagglutinin titers of all patients. There was a significant correlation between Ig G and Ig M titer in initial baseline (r^2^ = 0.51, *p* < 0.001). Preoperative baseline isoagglutinin titers were significantly higher in the patients with AKI than those without AKI (Ig G: 1:512 [1:64 to 1:1024] vs. 1:128 [1:32 to 1:512], *p* = 0.004; Ig M: 1:128 [1:64 to 1:512] vs. 1:64 [1:16 to 1:256], *p* = 0.001, respectively). Postoperative peak titers of Ig M and Ig G were also significantly higher in the patients with AKI than those without AKI (Ig G: 1:64 [1:16 to 1:256] vs. 1:4 [negative to 1:32], *p* < 0.001; Ig M: 1:32 [1:8 to 1:64] vs. 1:4 [negative to 1:16], *p* < 0.001, respectively). Postoperative daily tacrolimus blood trough levels are compared between the patients with and without AKI in Supplementary Figure S1. Daily tacrolimus trough levels were not significantly different between the patients with and without AKI.

The results of multivariable logistic regression analysis for post-transplant AKI are shown in Table 2. Baseline isoagglutinin titer of Ig M was identified as a significant predictor of AKI (odds ratio 1.06, 95% confidence interval [CI] 1.02 to 1.09, *p* = 0.010). Postoperative peak isoagglutinin titer of Ig M was also significantly associated with postoperative AKI (odds ratio 1.08, 95% CI 1.04 to 1.13, *p* < 0.001). Peak tacrolimus trough level was not identified as a significant risk factor (odds ratio 1.08, 95% CI 0.91 to 1.32, *p* = 0.420). The performance of our multivariable regression model was fairly good (area under the receiver-operating characteristic curve = 0.78, 95% CI, 0.71–0.84, Negelkerke’s R2 = 0.286). The calibration of our model was good (Hosmer-Lemeshow goodness-of-fit, chi-square = 16.32, *p* = 0.435).

Supplementary Table S2 shows the results of the multivariable generalized estimating equation model. Isoagglutinin titers of perioperative Ig G and Ig M were significant contributors to the development of AKI (Ig M: odds ratio 1.04, 95% CI 1.02 to 1.08, *p* < 0.001; Ig G: odds ratio 1.02, 95% CI 0.98 to 1.05, *p* = 0.125).

**Figure 2:**
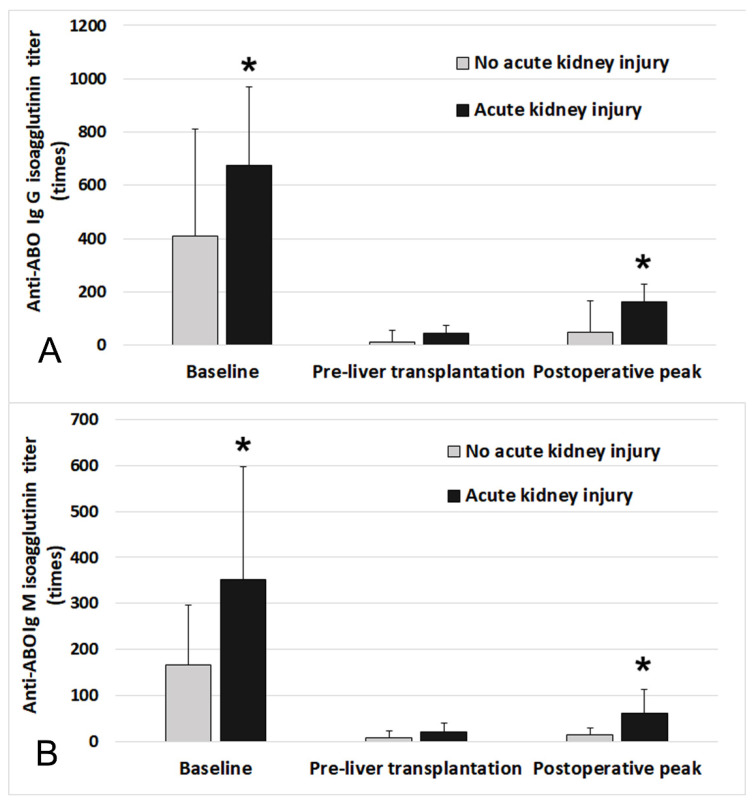
Anti-ABO blood type Ig G (**A**) and Ig M (**B**) isoagglutinin titers at baseline, pre-liver transplantation, and postoperative peak between patients with and without postoperative acute kidney injury. Pre-liver transplantation refers to the titer measured before the day of transplantation and postoperative peak titer was determined as the highest value during the postoperative two weeks. * Significant difference between groups (*p* < 0.05). Ig = immunoglobulin. The top edge of each box shows the mean value and the error bar shows standard deviation.

Figure 2 compares the time-dependent distribution of the Ig M and Ig G isoagglutinin titers at baseline, pre-LT, and postoperative peak between the patients with and without postoperative AKI. There was a significant difference in Ig M and Ig G titers at baseline and postoperative peak (baseline: Ig M, *p* = 0.005, Ig G, *p* = 0.008; postoperative peak: Ig M, *p* < 0.001, Ig G, *p* < 0.001).

**Figure 3:**
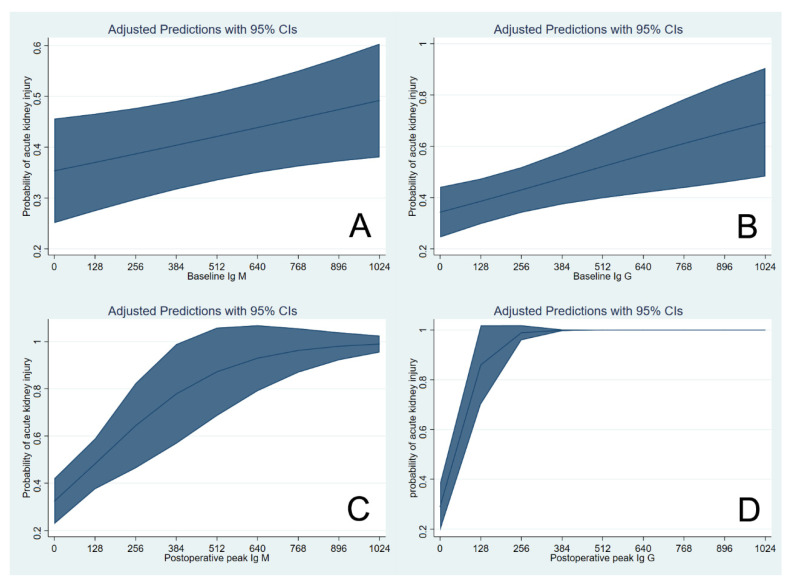
Cubic spline function curve relating initial baseline isoagglutinin titers of Ig M (**A**) and Ig G (**B**) and postoperative peak titers of Ig M (**C**) and Ig G (**D**) to the risk of acute kidney injury in ABO-incompatible liver transplantation. Ig = immunoglobulin. Shaded area represents 95% confidence interval of each estimated probability.

Figure 3 shows the cubic spline function curve to show the adjusted association of initial baseline and postoperative peak isoagglutinin titers with the risk of AKI. The cubic splines were positively sloped, and the slope was steeper for the postoperative peak Ig M isoagglutinin titers.

Sixteen patients (12.3%) did not reach the target titer of 1:16 and received splenectomy during transplantation surgery and postoperative IVIG. Table 3 shows the comparison of TPE-related parameters and clinical outcomes between patients with high and low initial baseline isoagglutinin groups. There was a significant difference in the incidence of AKI between the two groups (high: *n* = 29, 52.7%, low: *n* = 26, 10.2%, *p* = 0.021). The median number of TPE treatments was seven in the low initial baseline Ig M titer group and 11 in the high titer group. The incidence of post-LT TPE due to isoagglutinin rebound was significantly higher in the baseline high Ig M titer group than in the low titer group. However, other secondary outcomes, including the incidence of infection and surgical complication, were not significantly different between groups.

Table 4 compares the TPE-related parameters and clinical outcomes between the patients in high and low postoperative peak isoagglutinin groups. There was a significant difference in the incidence of AKI between the groups (high: *n* = 40, 67.8%, low: *n* = 15, 21.1%, *p* < 0.001). There was a significant difference in the incidence of postoperative hemodialysis between groups.

**Figure 4:**
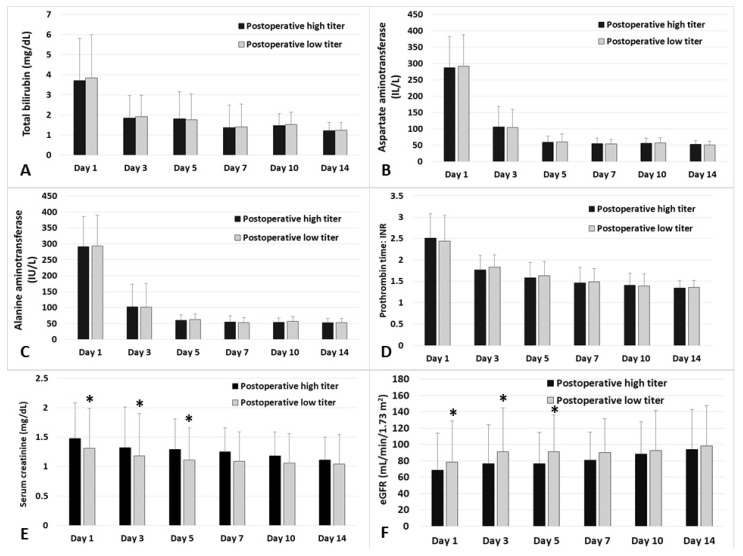
Laboratory results after liver transplantation between the high and low postoperative peak Ig M isoagglutinin titer groups (high group: peak Ig M titer ≥ 32; low group: peak Ig M titer ≤ 16). Laboratory values, including total bilirubin (**A**), aspartate aminotransferase (**B**), alanine aminotransferase (**C**), prothrombin time: international normalized ratio (**D**), serum creatinine (**E**), and estimated glomerular filtration rate (**F**) are depicted during postoperative day 1 to 14. * = significant difference between groups (*p* < 0.05); Ig = immunoglobulin; eGFR = estimated glomerular filtration rate. The top edge of each box shows the mean value, and the error bar shows standard deviation.

Figure 4 shows the daily comparison of postoperative laboratory values regarding liver graft and renal function until two weeks postoperative between the high and low postoperative peak Ig M isoagglutinin titer groups. There were significant differences in serum creatinine and estimated glomerular filtration rate on postoperative days 1, 3 and 5.

The risk of death or graft failure during five years after transplantation in the high baseline isoagglutinin titer group was not significantly different from the low baseline isoagglutinin titer group (hazard ratio 2.74, 95% CI 0.49 to 15.3, *p* = 0.250). The risk in the high postoperative peak isoagglutinin titer group was not significantly different from the low titer group (hazard ratio 2.40, 95% CI 0.47 to 12.2, *p* = 0.292). Supplementary Figure S2 shows the Kaplan-Meier survival curve analysis. There was no significant difference in the graft survival between the low and high baseline or postoperative peak isoagglutinin groups (log-rank test *p* = 0.631 and *p* = 0.862, respectively).

## 4. Discussion

The major finding of our retrospective analysis of our cohort of ABO-i LT is that the risk of postoperative AKI is significantly associated with the initial baseline and postoperative peak isoagglutinin titers of anti-ABO blood type antibodies. Multivariable logistic regression analysis and a generalized estimating equation model showed that baseline and immediate postoperative isoagglutinin titers of Ig M antibodies are significant predictors of the development of postoperative AKI. Although both baseline and postoperative peak Ig M antibodies are significant, postoperative peak Ig M isoagglutinin titer had the largest effect size in our regression model. However, we found that graft function-related laboratory findings and incidence of complications were not significantly different between the high and low isoagglutinin titer groups. Only the perioperative transfusion amount was significantly higher in the high isoagglutinin titer group, and the incidence of early allograft dysfunction tended to be higher in the high isoagglutinin titer group. These results suggest that the high risk of AKI in the high isoagglutinin titer group may be related to transfusion-associated renal injury and early allograft dysfunction. As the causal relationship between high isoagglutinin titer and AKI remains unclear, and our study was not adequately powered for these clinical outcomes, these results should be interpreted cautiously; high isoagglutinin titer can be regarded as a warning sign for high risk of postoperative AKI.

Graft survival and patient mortality after ABO-i LT are currently comparable to ABO-c LT [16,17,18,19]. However, we are still concerned about the high incidence of biliary stricture and antibody-mediated rejection associated with a high isoagglutinin titer [18]. Other major organ injury including AKI is also possible due to the high isoagglutinin titer through immunologic response [31]. Since AKI is associated with mortality and graft outcomes after liver transplantation [4,7,14], it would be relevant to investigate modifiable risk factors of AKI after ABO-i LT. However, there have been no previous studies reporting the association between perioperative isoagglutinin titers and post-transplant AKI.

The incidence of AKI after ABO-i LT in our study is relatively high but within the range reported in previous studies [1,3,5,6,8,9,10]. Previous studies have reported that AKI occurs more frequently in ABO-i LT [32,33]. The high isoagglutinin titer may be associated with this high incidence of AKI. A high isoagglutinin titer could produce liver graft damage via possible immunologic injury to the vascular endothelial cell [31]. Baseline isoagglutinin titer was significantly associated with the risk of AKI during the first postoperative week in our study, though TPE aimed to reduce the isoagglutinin titer to less than 1:8. This may be because not all patients reached the target range of low isoagglutinin titer, and postoperative rebound of isoagglutinin titer in these patients may further increase the risk. This was supported by our results of significant association of postoperative peak isoagglutinin titer with AKI during 2 weeks postoperative. Based on our study results, it would be helpful to mitigate the risk of AKI by delaying elective ABO-i LT until the target level of low isoagglutinin titer is reached and to perform timely postoperative additional TPE for patients whose isoagglutinin titer elevates postoperatively. It has been recommended that several post-transplant TPEs should routinely be received in all patients to prevent rebound of isoagglutinin titers until accommodation or tolerance is achieved [22].

However, the causal relationship between high baseline and postoperative peak isoagglutinin titer and the risk of AKI is unclear. Only the amount of transfusion was significantly higher and the incidence of early allograft dysfunction was higher in the ABO-i LT, suggesting that the high incidence of AKI could be related to the graft dysfunction or transfusion-associated inflammatory response. Our results should be interpreted cautiously because the high isoagglutinin titer and risk of AKI could be only an association. Another possible mechanism of renal injury is isoagglutinin-mediated hemolysis of passively transferred donor red blood cells in the liver graft. Free hemoglobin is toxic to kidneys, and this toxic effect could be significant to an already compromised kidney. However, we could not find any evidence of hemolysis in our data, such as high lactate dehydrogenase or low haptoglobin.

There could be other potential risk factors of AKI in ABO-i LT. The predictors of AKI after ABO-i LT found in our study were mostly consistent with previous studies. Preoperative hemoglobin level was significantly associated with post-transplant AKI [8,34,35]. Many previous studies reported that MELD score is a predictor of AKI [2,3,23]. A previous study reported that preoperative low platelet count, baseline metabolic alkalosis, and frequent mild arterial hypoxemia in ABO-i LT recipients could lead to higher requirements for perioperative transfusion and low oxygen delivery to kidney, resulting in a high risk of AKI [33]. Furthermore, the patients undergoing ABO-i LT receive a large amount of FFP transfusion for TPE before transplantation. FFP is a known predictor of AKI after liver transplantation [2,36,37], and the risk of AKI may further increase when patients receive intraoperative FFP transfusion. Our multivariable prediction model revealed that intraoperative transfusion amounts of pRBC and FFP are significantly associated with AKI. In addition, an allergic, inflammatory and immunologic reaction initiated by preoperative FFP transfusion may exacerbate when patients receive intraoperative FFP [38,39]. Transfusion compatible with graft ABO blood type is not always possible during the perioperative period in ABO-i LT. Therefore, transfusion-related immunologic and inflammatory reactions could be greater in ABO-i LT than ABO-c LT, and these adverse inflammatory responses may contribute to the development of AKI.

Red blood cell transfusion is significantly associated with post-transplant AKI [2,35]. The adverse impact of pRBC transfusion is known to be multifaceted [40,41] and includes systemic inflammatory response, which increases the risk of AKI after surgery. A significant amount of intraoperative transfusion is related to excessive surgical bleeding, which leads to systemic and renal hypoperfusion, resulting in poor oxygen delivery to the major organs. The adverse effect of intraoperative pRBC transfusion could be greater in patients undergoing ABO-i LT [31,32]. This could be associated with the high anti-ABO blood type isoagglutinin titer.

Our study has several important limitations. First, our study is a single-center retrospective study. Unknown or unmeasured covariates could affect our study results and external validity could be limited depending on the different protocols of desensitization and immunosuppression. The causal relationship between baseline and immediate postoperative isoagglutinin titer and AKI could not be revealed due to the retrospective design. Second, there was only a small sample size in our cohort of ABO-i LT. This limited the power of our multivariable analysis and analysis regarding less frequent secondary outcomes. Third, during the course of TPE, the nature and types of human leukocyte antigen (HLA) or non-HLA antibodies other than ABO blood-type antibodies could not be obtained due to lack of data. These data could affect the prognosis of liver grafts.

## 5. Conclusions

Our study demonstrated that the baseline and peak postoperative isoagglutinin titers of Ig G and Ig M blood type antibodies are associated with the risk of AKI after ABO-i living donor LT. Our cubic spline function analysis showed that there is a continuous positive relationship between preoperative baseline and peak postoperative isoagglutinin titers and the risk of AKI after ABO-i living donor LT. However, as the causal relationship between high isoagglutinin titers and risk of AKI is unclear, the high isoagglutinin titer could be used simply as a warning sign for the risk of AKI after liver transplantation. Additional caution could be used in patients with known baseline renal insufficiency and high baseline or postoperative isoagglutinin titers.

## Figures and Tables

**Table 1:** Comparison of patient demographics and characteristics between patients with high and low initial baseline Ig M isoagglutinin titers.

Characteristic	Baseline High Ig M Titer (≥1:128) (*n* = 55)	Baseline Low Ig M Titer (≤1:64) (*n* = 75)	*p*-Value
Age, year, median (range)	53 (49–59)	55 (50–62)	0.128
Male, n	44 (58.7)	37 (67.3)	0.317
Body-mass index, kg/m^2^	23.5 (22.0–26.3)	23.1 (20.9–25.4)	0.168
MELD score	13 (8–20)	11 (9–19)	0.803
Child class, n (A/B/C)	25 (45.5)/21 (38.2)/9 (16.4)	37 (49.3)/23 (30.7)/15 (20.0)	0.652
ABO blood group, recipient, n			<0.001
O/A/B	43 (78.2)/5 (9.1)/7 (12.7)	18 (24.0)/29 (38.7)/28 (37.3)	
ABO blood group, donor, n			0.492
A/B/AB	23 (41.8)/22 (40.0)/10 (18.2)	38 (50.7)/28 (37.3)/9 (12.0)	
Etiology of liver disease, n			
Alcoholic liver disease, n	8 (14.5)	11 (14.7)	0.985
HBV hepatitis, n	4 (7.3)	7 (9.3)	0.677
HCV hepatitis, n	1 (1.8)	2 (2.7)	0.999
Cholestatic disease, n	5 (9.1)	10 (13.3)	0.582
Non-alcoholic steatohepatitis, n	3 (5.5)	1 (1.3)	0.310
Hepatocellular carcinoma, n	34 (61.8)	44 (58.7)	0.717
Type of graft used (right vs. left lobe)	53 (96.4)/2 (3.6)	70 (93.3)/5 (6.7)	0.698
Graft-to-recipient body weight ratio	1.23 (1.12–1.48)	1.18 (1.09–1.43)	0.451
Cold ischemic time, min	82 (69–97)	81 (72–96)	0.280
Warm ischemic time, min	26 (19–31)	27 (23–32)	0.080
Baseline estimated glomerular filtration rate, mL/min/1.73 m^2^	92.6 (67.9–126.7)	93.1 (65.0–123.8)	0.459
Cross-matching prior to transplant (positive vs. negative)	1 (1.8)/54 (98.2)	2 (2.7)/73 (97.3)	0.750
Initial baseline Ig M titer	1:1024 (1:512–1:2048)	1:64 (1:16–1:128)	<0.001
Initial baseline Ig G titter	1:256 (1:128–1:512)	1:32 (1:16–1:64)	0.010
Final pre-LT Ig M titer	1:16 (1:8–1:32)	1:4 (negative–1:8)	0.011
Final pre-LT Ig G titer	1:8 (1:4–1:32)	1:2 (negative–1:8)	0.006
Postoperative peak Ig M titer	1:128 (1:16 to 1:256)	1:4 (negative–1:16)	0.004
Postoperative peak Ig G titer	1:32 (1:8 to 1:64)	1:4 (negative–1:16)	0.015

Data are presented as median (interquartile range) or number (%). Ig = immunoglobulin, MELD score = Model for end stage liver disease score, HBV = hepatitis B virus, HCV = hepatitis C virus, HCC = hepatocellular carcinoma, LT = liver transplantation.

**Table 2:** Multivariable logistic regression analysis for acute kidney injury after liver transplantation.

Variable	Odds Ratio	95% Confidence Interval	*p*-Value
Age, recipient	1.06	0.99–1.13	0.089
Body-mass index, recipient	1.08	1.01–1.26	0.042
MELD score	1.09	1.00–1.22	0.040
Preoperative hemoglobin, g/dL	0.86	0.65–1.13	0.181
Postreperfusion syndrome	1.16	0.81–1.24	0.075
Intraoperative pRBC transfusion, per unit	1.07	1.04–1.12	<0.001
Intraoperative FFP transfusion, per unit	1.06	1.02–1.11	<0.001
Peak tacrolimus trough level during postoperative seven days, ng/ml	1.08	0.91–1.32	0.420
Initial baseline Ig M titer	1.06	1.02–1.09	0.010
Initial baseline Ig G titter	1.02	0.99–1.05	0.159
Final pre-LT Ig M titer	1.00	0.98–1.02	0.859
Final pre-LT Ig G titer	1.02	0.97–1.04	0.112
Postoperative peak Ig M titer	1.08	1.04–1.13	<0.001
Postoperative peak Ig G titer	1.00	0.99–1.01	0.110

MELD score = model for end-stage liver disease score, pRBC = packed red blood cell, FFP = fresh frozen plasma, LT = liver transplantation, Ig = immunoglobulin.

**Table 3:** Comparison of plasma exchange-related parameters and post-transplant outcomes between patients with initial baseline high and low Ig M isoagglutinin titers.

Characteristic	Baseline High Ig M Titer (≥1:128) (*n* = 55)	Baseline Low Ig M Titer (≤1:64) (*n* = 75)	*p*-Value
Number of pre-LT TPE sessions, n	7 (6–8)	4 (2–6)	<0.001
Titer reduction rate	1.58 (1.18–2.12)	1.63 (1.24–2.02)	0.812
Post-LT TPE due to isoagglutinin titer rebound, n	37 (67.3%)	11 (14.7%)	<0.001
Antibody-mediated rejection due to anti-ABO antibody, n	-	-	-
Acute cellular rejection, n	3 (5.5%)	4 (5.3%)	0.976
Intraoperative estimated blood loss, mL	1800 (1100–3600)	1800 (1200–3200)	0.850
Perioperative transfusion amount, n			
Packed red blood cell	4 (0–10)	4 (0–9)	0.486
Fresh frozen plasma	4 (2–11)	3 (0–6)	0.048
Platelet, apheresis	0 (0–1)	0 (0–1)	0.567
IVIG with splenectomy, n	12 (21.8)	4 (5.3)	0.005
Acute kidney injury, during 1 week	29 (52.7%)	26 (34.7%)	0.021
Stage 1, n	16 (29.1%)	18 (24.0%)	
Stage 2 or 3, n	13 (23.6%)	8 (10.7%)	
Postoperative hemodialysis, n	9 (16.4%)	8 (10.7%)	0.341
Long-term hemodialysis (>6 months), n	2 (3.6)	1 (1.3)	0.573
Estimated glomerular filtration rate at 1 year after transplantation, mL/min/1.73 m^2^	91.4 (65.3–121.2)	92.5 (64.5–122.7)	0.459
Early allograft dysfunction, n	4 (7.3)	2 (2.7)	0.216
Length of hospital stay, days	19 (16–25)	18 (15–24)	0.356
Length of intensive care unit stay, days	5 (4–7)	5 (4–7)	0.561
Postoperative infection, n			
CMV/Bacterial/Fungal	5 (6.1%)/3 (5.5%)/0 (0%)	5 (6.7%)/4 (5.3%)/1 (1.3%)	0.390
Anastomotic biliary complication, n	3 (5.5%)	2 (2.7%)	0.414
Vascular complication, n	1 (1.8%)	2 (2.7%)	0.750
Reoperation, n	2 (3.6%)	1 (1.3%)	0.388

Data are presented as number (%) or median (interquartile range). Ig = immunoglobulin, LT = liver transplantation, TPE = therapeutic plasma exchange, IVIG = intravenous immunoglobulin. Titer reduction rate, which represents the mean titer reduction per one session of plasma exchange, was calculated as follows: TRR = (Isoagglutinin Titer Step Before Initiation of Plasma Exchange—Last Isoagglutinin Titer Step Before LT)/Number of Pre-KT Plasma Exchange.

**Table 4:** Comparison of plasma exchange-related parameters and post-transplant outcomes between patients with high and low postoperative peak Ig M isoagglutinin titers.

Characteristic	High Postoperative Peak Ig M Titer (≥1:32) (*n* = 59)	Low Postoperative Peak Ig M Titer (≤1:16) (*n* = 71)	*p*-Value
Number of pre-LT TPE sessions, n	8 (6–9)	5 (3–5)	<0.001
Titer reduction rate	1.58 (1.16–1.94)	1.71 (1.26–2.04)	0.378
Post-LT TPE due to isoagglutinin titer rebound, n	59 (100%)	-	
Antibody-mediated rejection due to anti-ABO antibody, n	-	-	-
Acute cellular rejection, n	4 (6.8%)	3 (4.2%)	0.701
Intraoperative estimated blood loss, ml	1600 (1100–3100)	1800 (1200–2900)	0.646
Perioperative transfusion amount, n			
Packed red blood cell	4 (0–10)	4 (0–9)	0.375
Fresh frozen plasma	4 (3–11)	3 (1–6)	0.040
Platelet, apheresis	0 (0–1)	0 (0–1)	0.657
IVIG with splenectomy, n	11 (18.6)	5 (7.0)	0.061
Acute kidney injury, during 1 week	40 (67.8%)	15 (21.1%)	<0.001
Stage 1, n	20 (33.9%)	14 (19.7%)	
Stage 2 or 3, n	20 (33.9%)	1 (1.4%)	
Postoperative hemodialysis, n	14 (23.7%)	3 (4.2%)	0.001
Long-term hemodialysis (>6 months), n	3 (5.1)	-	0.055
Estimated glomerular filtration rate at 1 year after transplantation, mL/min/1.73 m^2^	91.3 (67.2–123.6)	91.1 (67.7–124.4)	0.830
Early allograft dysfunction, n	5 (8.5)	1 (1.4)	0.056
Length of hospital stay, days	20 (14–27)	18 (15–22)	0.344
Length of intensive care unit stay, days	6 (5–8)	5 (4–7)	0.055
Postoperative infection, n			
CMV/Bacterial/Fungal	7 (11.9%)/5 (8.5%)/0 (0%)	3 (4.gh2%)/2 (2.8%)/1 (1.4%)	0.104
Anastomotic biliary complication, n	3 (5.1%)	2 (2.8%)	0.441
Vascular complication, n	2 (3.4%)	1 (1.4%)	0.590
Reoperation, n	2 (3.4%)	1 (1.4%)	0.590

Data are presented as number (%) or median (interquartile range). Ig = immunoglobulin, LT = liver transplantation, TPE = therapeutic plasma exchange, IVIG = intravenous immunoglobulin.

## Data Availability

The data presented in this study are available on request from the corresponding author.

## Supplementary Materials

The following are available online at https://www.mdpi.com/article/10.3390/jcm10081679/s1: Supplemental Table S1. Comparison of patient demographics and characteristics between patients with and without acute kidney injury. Supplemental Table S2. Generalized estimating equation model to evaluate the time-dependent association between the isoagglutinin titers, tacrolimus levels and postoperative risk of AKI. Supplemental Figure S1. Comparison of daily tacrolimus trough level (ng/mL) between the patients who developed acute kidney injury (AKI) and who did not. POD = postoperative days. Supplemental Figure S2. Kaplan-Meier survival curve analysis between the high and low baseline (A) and postoperative peak isoagglutinin groups (B).
