# Supplementary material for: Perioperative ABO Blood Group Isoagglutinin Titer and the Risk of Acute Kidney Injury after ABO-Incompatible Living Donor Liver Transplantation
Source: J Clin Med. 2021 Apr 14;10(8):1679. doi: 10.3390/jcm10081679 (PMC8070732; doi:10.3390/jcm10081679)
Supplement: Supplementary file 1 [file jcm-10-01679-s001.pdf]

Journal of Clinical Medicine

Perioperative ABO Blood Group Isoagglutinin Titer and the Risk of Acute Kidney Injury after ABO-Incompatible Liver Transplantation

Supplemental Materials

Table S1. Comparison of patient demographics and characteristics between patients with and without acute kidney injury.

Characteristic	No AKI (*n*=75)	AKI Stage 1 (*n*=34)	AKI Stage 2 or 3 (*n*=21)	*p*-Value
Age, year, median (range)	55 (50 – 61)	56 (49 – 69)	58 (53 – 68)	0.059
Male, *n*	43 (57.3)	26 (76.5)	12 (57.1)	0.140
Body-mass index, kg/m²	23.1 (20.9 – 25.5)	23.3 (22.0 – 26.1)	23.5 (22.2 – 26.5)	0.022
MELD score	10 (8 – 14)	18 (13 – 20)	22 (17 – 24)	<0.001
Child class, *n* (A/B/C)	41 (54.7)/ 25 (33.3)/ 9 (12.0)	14 (41.2)/ 12 (35.3)/ 8 (23.5)	7 (33.3)/ 7 (33.3)/ 7 (33.3)	0.153
ABO blood group, recipient, *n*				
O/ A/ B	30 (40.0)/ 21 (28.0)/ 24 (32.0)	20 (58.8)/ 9 (26.5)/ 5 (14.7)	11 (52.4)/ 4 (19.0)/ 6 (28.6)	0.452
ABO blood group, donor, *n*				
A/ B/ AB	33 (44.0)/ 32 (42.7)/ 10 (13.3)	18 (52.9)/ 10 (29.4)/ 6 (17.6)	10 (47.6)/ 8 (38.1)/ 3 (14.3)	0.778
Etiology of liver disease, *n*				
Alcoholic liver disease, *n*	10 (13.3)	5 (14.7)	4 (19.0)	0.807
HBV hepatitis, *n*	4 (5.3)	4 (11.8)	3 (14.3)	0.136
HCV hepatitis, *n*	2 (2.7)	1 (2.9)	-	0.560
Cholestatic disease, *n*	8 (10.7)	3 (8.8)	4 (19.0)	0.417
Non-alcoholic steatohepatitis, *n*	2 (2.7)	1 (2.9)	1 (4.8)	0.656
HCC, *n*				
HCC associated with HBV hepatitis , *n*	44 (58.7)	19 (55.9)	8 (38.1)	0.129
HCC associated with HCV hepatitis, *n*	5 (6.7)	1 (2.9)	1 (4.8)	0.574
Initial baseline Ig M titer	1:64 (1:16 – 1:512)	1:256 (1:256 – 1:1280)	1:1024 (1:224 – 1:2048)	<0.001
Initial baseline Ig G titter	1:32 (1:16 – 1:128)	1:256 (1:64 – 1:512)	1:256 (1:64 – 1:521)	<0.001
Final pre-LT Ig M titer	1:4 (none – 1:8)	1:16 (1:4 – 1:32)	1:16 (1:4 - 1:96)	<0.001
Final pre-LT Ig G titer	1:2 (none – 1:4)	1:8 (1:4 – 1:16)	1:8 (1:4 – 1:24)	<0.001
Postoperative peak Ig M titer	1:4 (none – 1:32)	1:64 (1:16 – 1:128)	1:128 (1:16 – 1:256)	<0.001
Postoperative peak Ig G titer	1:4 (none – 1:16)	1:24 (1:8 – 1:36)	1:32 (1:10 – 1:96)	<0.001

Data are presented as median (interquartile range) or number (%). AKI = acute kidney injury, Ig = immunoglobulin, MELD score = Model for end stage liver disease score, HBV = hepatitis B virus, HCV = hepatitis C virus, HCC = hepatocellular carcinoma, LT = liver transplantation.

Table S2. Generalized estimating equation model to evaluate the time-dependent association between the isoagglutinin titers, tacrolimus levels and postoperative risk of AKI.

Variable	Odds Ratio	95% CI	*p*-Value
Age, recipient	1.05	0.98 – 1.12	0.101
Body-mass index, recipient	1.09	1.00 – 1.22	0.045
MELD score	1.10	1.01 – 1.20	0.030
Preoperative hemoglobin, g/dL	0.85	0.63 – 1.11	0.081
Intraoperative pRBC transfusion, per unit	1.06	1.03 – 1.15	<0.001
Intraoperative FFP transfusion, per unit	1.05	1.02 – 1.13	0.001
Tacrolimus trough level during postoperative seven days, ng/ml	1.09	0.92 – 1.35	0.357
Perioperative Ig M titer	1.04	1.02 – 1.08	<0.001
Perioperative Ig G titter	1.02	0.98 – 1.05	0.125

MELD score = model for end-stage liver disease score; pRBC = packed red blood cell; FFP = fresh frozen plasma; Ig = immunoglobulin.

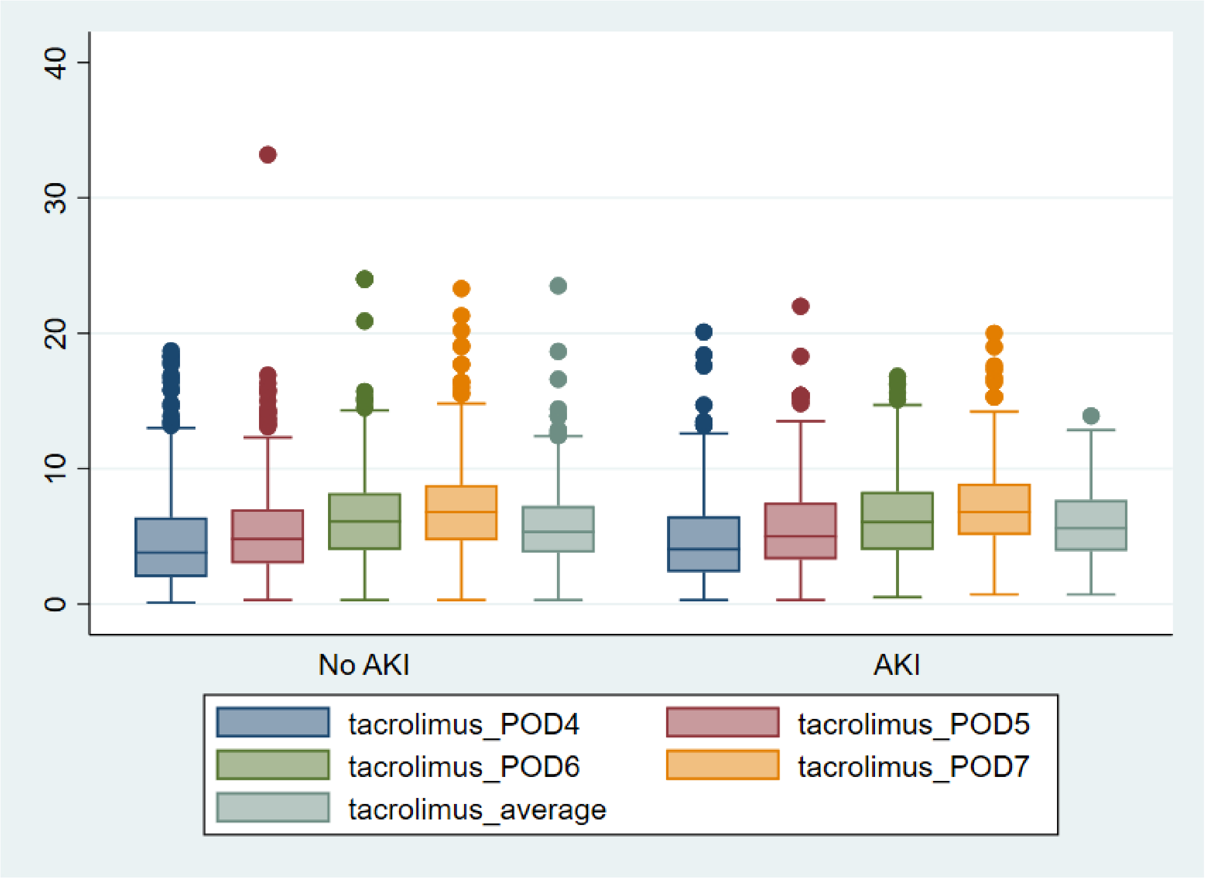

Figure S1. Comparison of daily tacrolimus trough level (ng/mL) between the patients who developed acute kidney injury (AKI) and who did not. POD = postoperative days.

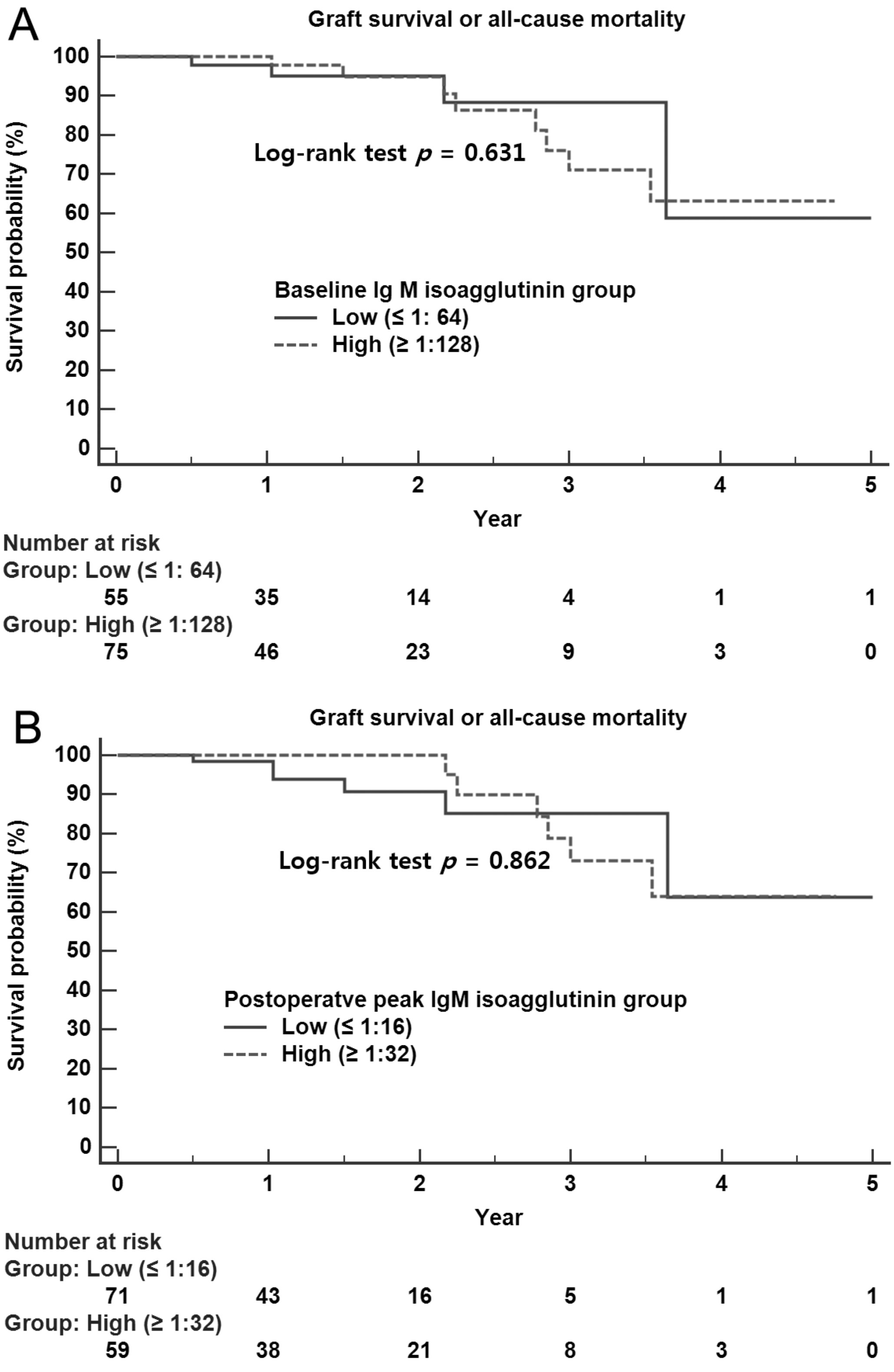

Figure S2. Kaplan-Meier survival curve analysis between the high and low baseline (**A**) and postoperative peak isoagglutinin groups (**B**).
